# Molecular basis for the different PCV2 susceptibility of T-lymphoblasts in Landrace and Piétrain pigs

**DOI:** 10.1186/s13567-024-01275-0

**Published:** 2024-02-19

**Authors:** Yueling Ouyang, Hans J. Nauwynck

**Affiliations:** Laboratory of Virology, Department of Translational Physiology, Infectiology and Public Health, Faculty of Veterinary Medicine, Ghent University, Ghent, Belgium

**Keywords:** PCV2, PCVAD, Landrace, Piétrain, T-lymphoblasts, replication, glycosaminoglycans, endo-lysosomes, low-pH, viral escape

## Abstract

Clinically, Landrace pigs are more susceptible to porcine circovirus-associated diseases (PCVADs) than Piétrain pigs. We previously found that porcine circovirus type 2 (PCV2) can infect T-lymphoblasts. The present study examined the replication kinetics of six PCV2 strains in the lymphoblasts of Landrace and Piétrain pigs. The results showed that T-lymphoblasts from Landrace pigs are much more susceptible to PCV2 infection than those from Piétrain pigs. In addition, PCV2 replication was strain-dependent. PCV2 binding to T-lymphoblasts was partially mediated by chondroitin sulfate (CS) and dermatan sulfate (DS). Phosphacan, an effective internalization mediator in monocytes that contains several CS chains, was also demonstrated to be involved in PCV2 internalization. Viral binding and internalization were not different between the two breeds, however, the subsequent step, the disassembly was. Although inhibition of serine proteases blocked PCV2 replication in both Landrace and Piétrain pigs, this only occurred at a neutral pH in Piétrain pigs, whereas this occurred also at a low pH in Landrace. This suggested that more proteases can cleave PCV2 in Landrace lymphoblasts than in Piétrain lymphoblasts, explaining the better replication. Through co-localization studies of viral particles with endo-lysosomal markers, and quantitative analysis of organelle sizes during viral internalization, it was observed that PCV2 may exhibit a higher propensity for viral escape from late endosomes in Landrace pigs (smaller) compared to Piétrain pigs. These results provide new understandings of the different PCV2 susceptibility in Landrace and Piétrain pigs.

**Supplementary Information:**

The online version contains supplementary material available at 10.1186/s13567-024-01275-0.

## Introduction

Porcine circovirus type 2 (PCV2) is a small, non-enveloped virus with single-stranded, closed-circular DNA, classified in the genus *Circovirus* of the family *Circoviridae* [1–3]. PCV2 is the causative agent of Porcine circovirus associated diseases (PCVADs) in pigs globally. These diseases encompass a spectrum of clinical manifestations, including postweaning multisystemic wasting syndrome (PMWS), reproductive failure, porcine dermatitis and nephropathy syndrome (PDNS), porcine respiratory disease complex (PRDC), enteritis, congenital tremor (CT), and proliferative and necrotizing pneumonia (PNP), among other pathological conditions [4–6]. The genome of PCV2 is comprised of 1766 to 1768 nucleotides and primarily encodes two open reading frames (ORFs) [7]. ORF1 encodes the replicase protein (Rep), a key component involved in viral replication [8, 9]. ORF2 encodes the capsid protein (Cap), which constitutes the only structural protein [10].

In pigs with PCVADs, lymphoid tissues show histopathological lesions, including monocyte infiltration and lymphocyte depletion [11–13]. Although PCV2 can target multiple cell types, not all support efficient viral replication. The virus relies on cellular polymerase, primarily found in mitotic cells, for facilitating the replication of its genomic material [14, 15]. Compared to the non-productive infection in monocyte/macrophage lineage cells, proliferating lymphoblasts are vital targets for PCV2 replication [15–17]. Landrace pigs infected with PCV2 have been reported to show a higher susceptibility to PCV2-associated lesions and PMWS development compared to Piétrain pigs [18, 19]. The varying susceptibility to PCV2 infection among pig breeds suggests the involvement of host-specific factors influencing viral replication and pathogenesis.

In our previous research, we established that PCV2 successfully replicates in T-lymphoblasts and enters cells via clathrin-mediated endocytosis [20]. Many viruses, including those entering host cells via clathrin-mediated endocytosis, undergo intracellular trafficking via the endo-lysosomal pathway. Viral entry often leads to two possible outcomes: successful escape en route or ultimate degradation within the lysosome, a cellular dead end.

Regarding PCV2, the fate of viral particles within the endo-lysosomal system may significantly affect the susceptibility of T-lymphoblasts and disease outcomes across different pig breeds and different PCV2 strains. Therefore, we examined the behavior of different PCV2 strains in T-lymphoblasts of Piétrain and Landrace pigs, in order to uncover the relationship between PCV2 susceptibility and the viral behavior within the endo-lysosomal system, and tried to elucidate the underlying mechanisms.

## Materials and methods

### Animals and virus stocks

In this study, 9 purebred Landrace pigs and 6 purebred Piétrain pigs, with ages ranging from 6 to 13 weeks, were used as blood donors. The pigs were housed individually throughout the study period. Six distinct PK-15-grown PCV2 strains were used due to their representation in PCV2 evolution and pathogenicity. More detailed information can be found in our previous study [21]. Strain Stoon-1010 (PCV2a; GenBank accession #AAC35310.1) was isolated from PMWS-affected piglets in Canada in 1998 [22], had a titer of 10^5.8^ TCID_50_/mL and an in vitro passage of 24. Strain 1121 (PCV2a; #AJ293868.1) was isolated from aborted fetuses in Canada in 2001 [23], had a titer of 10^4.96^ TCID_50_/mL and an in vitro passage of 26. Strain 1147 (PCV2b; #AJ293869.1) originated from a case of PDNS in the UK in 2001 [23], had a titer of 10^5.3^ TCID_50_/mL and an in vitro passage of 10. Strain 09V448 (PCV2d-1; #QCQ78431.1) was obtained from the lymph nodes of a pig with a high PCV2 viral load (PCVAD^high^) during a PCVAD outbreak in Belgium in 2009 [24], had a titer of 10^5.3^ TCID_50_/mL and an in vitro passage of 5. Strain DE222-13 (PCV2d-2; #AJP07089.1) was isolated from a pig during a PCVAD^high^ outbreak in Germany in 2015 [25], had a titer of 10^4.96^ TCID_50_/mL and an in vitro passage of 9. Strain 19V245 (PCV2d-2; #USV27802.1) originated from the lymph nodes of a pig with a PCVAD^high^ in Belgium in 2019 [21], had a titer of 10^5.6^ TCID_50_/mL and an in vitro passage of 6. To remove large viral clusters and cellular debris, all virus stocks were filtered through 0.45 µm MF-Millipore membrane filters.

### Antibodies and reagents

The antibodies and main reagents used, along with their dilutions, are listed in Tables 1 and 2, respectively.

**Table 1:** **Information of antibodies used in this study**

Antibody name	Class	Host	Mono/poly-clonal	Dilution for IF	Distributor	Catalog number
Anti-PCV2-Cap	IgG2b	Mouse	Mono	1:50	Made in our lab	–
Anti-PCV2-Rep	IgG	Rabbit	Poly	1:500	GeneTex; Irvine, CA, USA	GTX133638
Anti-CD3	IgG1	Mouse	Mono	1:100	Bio-Rad; Hercules, CA, USA	MCA5951GA
Anti-HS	IgM	Mouse	Mono	1:50	Amsbio; Abingdon, OX, UK	370255
Anti-CS	IgM	Mouse	Mono	1:50	Sigma-Aldrich; St. Louis, MO, USA	C-8035
Anti-decorin	IgG	Rabbit	Poly	1:50	GeneTex; Irvine, CA, USA	GTX101250
Anti-PTPRZ	IgG	Rabbit	Poly	1:50	Abbexa; Cambridge, CB, UK	abx131519
Anti-Rab5	IgG	Rabbit	Poly	1:50	Proteintech; Rosemont, IL, USA	11947-1-AP
Anti-Rab7	IgG	Rabbit	Poly	1:50	Cusabio; Houston, TX, USA	CSB-PA019219LA01HU
Anti-LAMP1	IgG	Rabbit	Poly	2:50	Proteintech; Rosemont, IL, USA	21997-1-AP

All relevant secondary antibodies used in this study were all purchased from Invitrogen (Thermo Fisher Scientific, Waltham, MA, USA). The incubation of secondary antibodies was performed and optimized according to the instructions of the manufacturers.

**Table 2:** **List of main reagents used in this study**

Reagent	Abbreviation	Distributor	Catalog Number
Ficoll-Paque plus	–	Cytiva; Marlborough, MA, USA	GE17-1440-03
RPMI-1640 medium	–	Gibco; Billings, MT, USA	72400021
Fetal calf serum	FCS	Gibco; Billings, MT, USA	16010159
Concanavalin A	Con A	Sigma-Aldrich; St. Louis, MO, USA	C5275
Interleukin-2 human	IL-2	Sigma-Aldrich; St. Louis, MO, USA	I7908
β-mercaptoethanol	β-ME	Gibco; Billings, MT, USA	31350010
Cell Counting Kit 8	CCK-8	Abcam; Cambridge, CB, UK	ab228554
Chloroquine	CQ	Sigma-Aldrich; St. Louis, MO, USA	C6628
4-(2-aminoethyl) benzenesulfonyl fluoride	AEBSF	Sigma-Aldrich; St. Louis, MO, USA	A8456
Cathepsin G inhibitor I	CatG inhibitor I	Sigma-Aldrich; St. Louis, MO, USA	219372

### PBMC isolation and T-lymphoblast generation

Porcine T-lymphoblasts were generated as described previously [20]. Briefly, peripheral blood mononuclear cells (PBMCs) were isolated using density gradient centrifugation on Ficoll-Paque, serving as the initial step for generating porcine T-lymphoblasts. The PBMCs were then cultured overnight in RPMI-1640 medium supplemented with 5% fetal calf serum (FCS) and antibiotics at 37 °C with 5% CO_2_. After being collected from PBMCs, the non-adherent lymphocytes were stimulated with 5 μg/mL concanavalin A (ConA) in a 10% leukocyte culture medium to promote T-lymphocyte activation. Following three days of incubation, the cells were maintained in fresh culture medium containing 50 U/mL of human recombinant interleukin-2 (IL-2) to further support T-lymphoblast growth. During proliferation, the expanded cells were transferred to more wells. This finally enabled the harvesting of proliferating cells, primarily T-lymphoblasts [12, 20].

### Quantification of viral particles

Quantification of PCV2 particles was performed following the method described in an earlier study from our research group [21]. In this study, red fluorescent microspheres were used to visualize the PCV2 particles, as illustrated in Additional file 2.

### Replication kinetics of different PCV2 strains in T-lymphoblasts

T-lymphoblasts were inoculated with different PCV2 strains at a MOI of 0.05 at 37 °C for 1 h. Subsequently, the cells were washed twice with ice-cold RPMI-1640, resuspended in fresh culture medium, and seeded at a concentration of 3 × 10^5^ cells/mL in 24-well cell culture plates. At different time points (0 h, 12 h, 24 h, 36 h, 48 h, and 72 h), both the supernatant and cells were collected for further analysis.

Supernatant fluids were titrated using immunoperoxidase monolayer assay (IPMA) as previously described [24, 26]. Cell smears were prepared on microscope slides using a Cytospin centrifuge, followed by fixation with 4% paraformaldehyde (PFA) for 10 min at room temperature (RT). Subsequently, the cells were permeabilized with 0.1% Triton X-100 for 2 min at RT. A double immunofluorescence (IF) staining was conducted to detect the expression of Rep proteins and Cap proteins. Rep and Cap were stained with rabbit anti-Rep polyclonal antibody (pAb) (IgG) and mouse anti-Cap monoclonal antibody (mAb) 12E12 (IgG2b), respectively, followed by the conjugated corresponding secondary Abs. Cellular nuclei were counterstained with Hoechst 33342 for 10 min. Cells were then dried and mounted using a glycerol solution containing 1,4-diazabicyclo octane (DABCO) anti-fading agents. Fluorescent images were captured with a Leica TCS SPE laser-scanning confocal microscope (LSCM; Leica, Germany). To quantify the percentages of PCV2 Rep^+^ or Cap^+^ cells, 10 randomly chosen areas were analyzed from each slide.

### Internalization of PCV2 particles into T-lymphoblasts

T-lymphoblasts were inoculated with 10^9^ PCV2 particles at a concentration of 3 × 10^5^ cells/mL at 37 °C for 60 min. After discarding the virus inoculum, cells were washed twice with warm RPMI-1640 medium. Afterwards, cells were fixed with 4% PFA for 10 min at RT and then smeared onto microscope slides using a cytospin. After washing, cells were permeabilized with 0.1% TritonX-100 for 2 min at RT. A double IF staining was performed to visualize both viral particles and cells. T-lymphoblasts were stained with mouse anti-CD3 mAb (IgG1), and PCV2 capsid proteins were stained with mouse anti-Cap mAb 12E12 (IgG2b), followed by conjugated corresponding secondary Abs. Nuclei were co-stained with Hoechst 33342 as previously described, and samples were mounted and analyzed under the LSCM. 10 random images were captured from each slide to quantify the percentage of cells with internalized viral particles and the Cap fluorescence pixels per PCV2^+^ cell using MATLAB software.

### Expression of glycosaminoglycans (GAGs) and phosphacan on T-lymphoblasts

As reported in a previous study, heparan sulfate (HS), chondroitin sulfate (CS), and decorin participate to varying degrees in the uptake of PCV2 particles by blood monocytes. Phosphacan also serves as an effective mediator for PCV2 internalization into monocytes [21]. In this study, a series of IF stainings were performed to investigate the presence of various GAGs and phosphacan on T-lymphoblasts and their potential role in PCV2 entry. Cells were smeared onto microscopic slides after 4% PFA fixation, and the Abs targeting on HS, CS, decorin (for DS detection) and phosphacan were applied. PK-15 cells and HepG2 cells, known to express GAGs and phosphacan respectively, were used as positive controls and stained in parallel [21, 27]. The expression of these membrane proteins was determined with LSCM. The percentage of GAG^+^ and phosphacan^+^ cells, as well as the mean fluorescing area (pixels) per cell, were quantified from 10 randomly selected regions.

### Confocal microscopy for co-localization assay

The co-localization between PCV2 particles and relevant membrane molecules (GAGs or phosphacan) was determined by confocal microscopy. 3 × 10^5^ cells were inoculated with 10^9^ virus particles and incubated for 60 min at 4 °C. The viral inoculum was removed before fixation with 4% PFA. Then, cells were smeared onto microscopic slides using a cytospin. A double IF staining was conducted to visualize both viral particles and GAGs. The attached PCV2 particles were stained with mouse anti-Cap 12E12 mAb, and GAGs/phosphacan were stained with specific anti-GAG/phosphacan Abs as described in Table 1. The percentage of cells displaying co-localization, and the fluorescence pixels related to PCV2 capsid proteins, GAG/phosphacan expression, and co-localization, were individually quantified from 10 randomly chosen confocal images using MATLAB software.

### Cytotoxicity tests

Cell Counting Kit 8 (CCK-8) assays were used to determine the cytotoxicity of chloroquine (CQ), 4-(2-Aminoethyl) benzenesulfonyl fluoride hydrochloride (AEBSF) and cathepsin G (CatG) inhibitor I on T-lymphoblasts. The kit assesses the cell viability by detecting the orange formazan dye using water-soluble tetrazolium salt. T-lymphoblasts were seeded at a density of 2 × 10^6^ cells/mL in 96-well plates. They were respectively treated with various concentrations of CQ (0, 1, 10, 25, 50 or 100 μM), AEBSF (0, 0.1, 0.2, 0.3 or 0.4 mM) or CatG inhibitor I (0, 0.1, 1, 10 or 100 μM) for 24 h at 37 °C in a 5% CO_2_ environment. 10 μL of CCK-8 solution was added to each well and incubated at 37 °C for 2 h. Absorbance was measured at 450 nm using a multi-plate reader. Cell viability was quantified as a percentage relative to the control group (cells without drug treatment).

### Effect of CQ, AEBSF and CatG inhibitor I on PCV2 infection to T-lymphoblasts

T-lymphoblasts were pre-treated at a density of 5 × 10^5^ cells/mL with different concentrations of drugs. Cells were incubated with different drugs under the following conditions: (i) CQ at concentrations of 0, 1, 10, 25, or 50 μM for 2 h at 37 °C, (ii) AEBSF at concentrations of 0, 0.1, or 0.2 mM for 1 h at 37 °C, and (iii) CatG inhibitor I at concentrations of 0, 0.1, 1, or 10 μM for 1 h at 37 °C. These treatments were paralleled by a cytotoxicity assay to ensure that cell viability remained above 80% (for CQ), 75% (for AEBSF), and 90% (for CatG inhibitor I), respectively. Afterwards, the cells were inoculated with either PCV2 strain 1121 or DE222-13 at 0.05 MOI, in the presence of the aforementioned drugs, at 37 °C for 1 h. After washing, cells were further cultured with CQ or CatG inhibitor I for 24 h at 37 °C. In the case of AEBSF, cells were exposed to the drug at 37 °C for 16 h, followed by incubation in a fresh medium without the drug for an additional 8 h. Subsequently, cells were fixed and permeabilized as described above. PCV2^+^ cells were determined by IF staining with a mouse anti-PCV2 Cap (12E12, IgG2b) mAb. The percentage of infected cells was quantified to assess PCV2 infection.

### PCV2 trafficking in T-lymphoblasts and organelle size analysis

A time-course experiment was conducted at intervals of 0, 5, 10, 20, 40, 60, 120, 180, and 360 min. At each time point, cells were fixed with 4% PFA and permeabilized with 0.1% TritonX-100. Subsequently, double IF stainings were conducted against various endosomal markers and PCV2 capsid proteins. Cells were stained with rabbit anti-Rab5, Rab7, or LAMP1 pAb to visualize EEs, LEs, and lysosomes, respectively. PCV2 capsid proteins were stained as described earlier to visualize viral particles. Co-localization between endosomal markers and PCV2 Cap proteins was detected using confocal microscopy, and the percentage of PCV2^+^ cells exhibiting co-localization with each marker was calculated. The area of co-localization between the markers and Cap, measured in pixels, was quantified using MATLAB software.

To assess changes in organelle sizes (EEs, LEs, and lysosomes) after viral internalization, the diameters of Rab5, Rab7, and LAMP1 were measured at specific time points (5 min, 10 min and 120 min) using Leica Application Suite X software. Additionally, the respective pixel sizes of the organelles were quantified using MATLAB software.

### Statistical analysis

Data are presented as mean ± standard deviation (SD). Statistical analysis was performed using GraphPad Prism 9 (GraphPad, USA) software with an unpaired Student *t*-test. Quantification of cells and fluorescent pixels was conducted using ImageJ (National Institutes of Health, USA) and MATLAB (MATrix LABoratory, USA) software. For all analysis, statistical significance was defined as *p* < 0.05 (*,* p* < 0.05).

## Results

### PCV2 replication in T-lymphoblasts varies within pig breeds and virus strains

**Figure 1:**
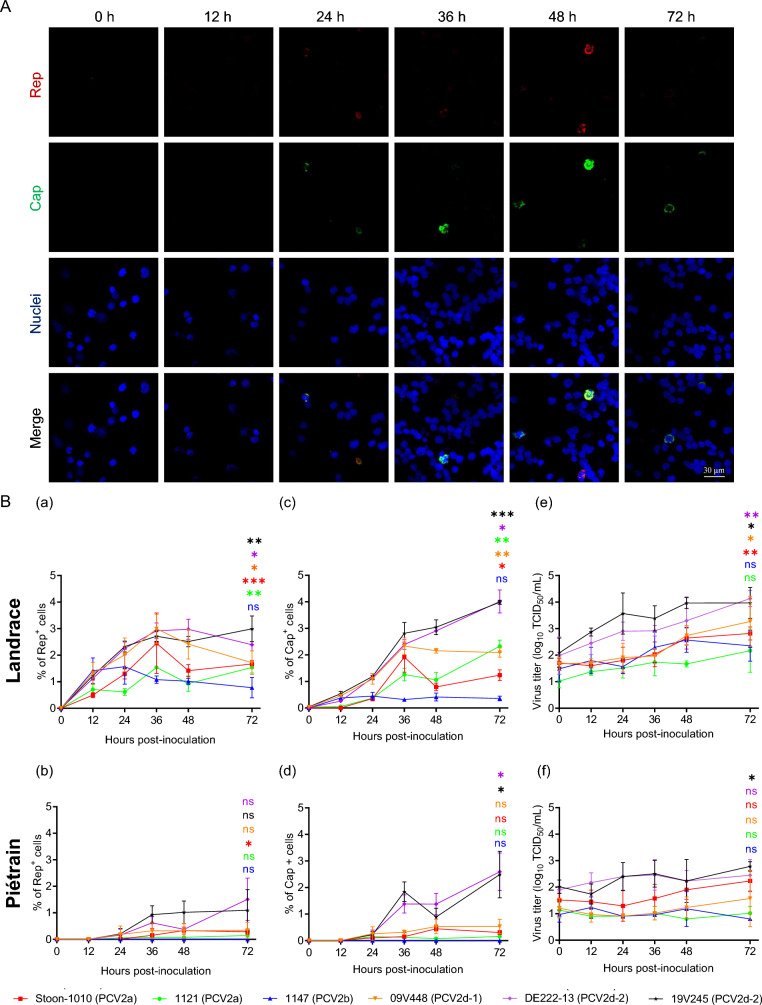
**PCV2 replication kinetics in T-lymphoblasts from Landrace and Piétrain pigs.** Cells were inoculated with six PCV2 strains [1121 (PCV2a), Stoon-1010 (PCV2a), 1147 (PCV2b), 09V448 (PCV2d-1), DE222-13 (PCV2d-2) and 19V245 (PCV2d-2)] at a MOI of 0.05 for 1 h at 37 °C. The data represent the mean values from three independent replicates (three pigs of each breed). Within each replicate, the mean was calculated based on ten randomly selected fields. **A** Representative confocal images showing strain DE222-13 replication in Landrace T-lymphoblasts. **B** The expression of Rep and Cap proteins was analyzed by IF staining to quantify the percentage of Rep^+^ cells (a, b) and Cap^+^ cells (c, d). The titer was determined via viral titration in PK-15 cells (e, f). Data represent the means ± SD of three replicates. Statistical difference was demonstrated between 1 and 72 h for each strain by the Student’s *t*-test post-Welch’s correction. *P < 0.05, **P < 0.01, ***P < 0.001.

T-lymphoblasts have been shown to support PCV2 replication. In vitro, T-lymphocytes exhibit significant proliferation when stimulated with Concanavalin A (ConA) and subsequently cultured with IL-2. As shown in Figure 1B, the percentage of PCV2^+^ cells displayed distinct kinetic profiles across different viral strains. In Landrace pigs, both Rep and Cap proteins were detectable as early as 12 h post-infection for each strain. However, until 36 h, during the first replication cycle, the synthesis of Rep consistently surpassed that of Cap, followed by a general decline until 72 h. Notably, the virulent PCV2d-2 strains, DE222-13 and 19V245, exhibited a continuous increase in Cap expression after 36 h. In contrast, the Cap detection of PCV2a strain Stoon-1010 and PCV2d-1 strain 09V448 peaked at 36 h and subsequently declined, entering the second replication cycle. PCV2a strain 1121 demonstrated a significantly higher Cap level (*p* = 0.0326) at the end of the second replication cycle (72 h) (2.3%) compared to 36 h (1.3%). PCV2b strain 1147 exhibited the lowest detection levels of Cap, remaining below 0.4% throughout the entire experimental period. In Piétrain pigs, Rep and Cap were detected from 24 h onwards for all strains except 1147. Strain 1147 remained undetectable throughout the entire duration, suggesting its inability to replicate in Piétrain pigs. Apart from the PCV2d-2 strains DE222-13 and 19V245, the level of Rep and Cap detection was consistently low for the other four strains. At 36 h, the expression levels of Rep varied among the strains in Landrace pigs: Stoon-1010 (2.5%), 1121 (1.5%), 1147 (1.1%), 09V448 (3.0%), DE222-13 (3.0%), and 19V245 (2.8%). In Piétrain pigs, the corresponding Rep expression levels were lower: Stoon-1010 (0.2%), 1121 (0.1%), 1147 (0), 09V448 (0.3%), DE222-13 (0.6%), and 19V245 (0.8%). Regarding Cap, the detections in Landrace pigs were: Stoon-1010 (1.9%), 1121 (1.3%), 1147 (0.3%), 09V448 (2.3%), DE222-13 (2.4%), and 19V245 (2.4%). In Piétrain pigs, the levels were significantly lower: Stoon-1010 (0.2%), 1121 (0.1%), 1147 (0), 09V448 (0.3%), DE222-13 (1.4%), and 19V245 (1.4%).

Cell culture supernatant was collected for virus titration. In Landrace pigs, the extracellular viral titers showed a gradual increase over time. Particularly, for the PCV2d-2 strains DE222-13 and 19V245, the titers increased by approximately 100-fold from 1 to 72 h (for DE222-13 from 10^2.0^ TCID_50_/mL to 10^4.1^ TCID_50_/mL; for 19V245 from 10^2.1^ TCID_50_/mL to 10^4.0^ TCID_50_/mL. The remaining four strains demonstrated moderate growth in titers throughout the entire duration (for strain 09V448 from 10^1.7^ TCID_50_/mL to 10^3.3^ TCID_50_/mL (40 ×); for Stoon-1010 from 10^1.7^ TCID_50_/mL to 10^2.8^ TCID_50_/mL (13 ×); for 1121 from 10^1.0^ TCID_50_/mL to 10^2.2^ TCID_50_/mL (14 ×), and 1147 from 10^1.5^ TCID_50_/mL to 10^2.4^ TCID_50_/mL (7 ×). However, for Piétrain pigs, the virus titers for all strains did not significantly increase.

Overall, the PCV2 replication in T-lymphoblasts from Landrace and Piétrain pigs is strain-dependent, with Landrace pigs exhibiting a higher efficiency in viral replication.

### PCV2 binding and internalization levels are similar in T-lymphoblasts from Landrace and Piétrain pigs

After ConA and IL-2 treatment, the obtained cells were mainly T-lymphoblasts. The proportion of CD3 (a T cell marker)-positive cells was quantified and compared between Landrace (90.6%) and Piétrain (85.2%) pigs, as shown in Additional file 3. It was found to be similar between the two breeds.

**Figure 2:**
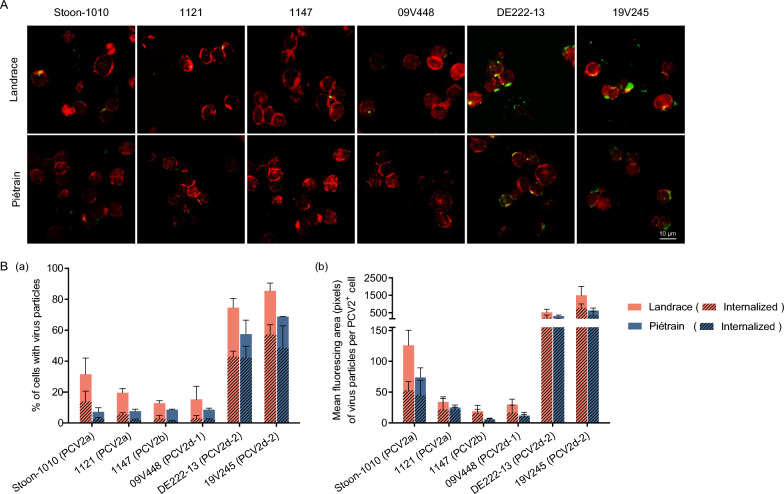
**PCV2 binding and internalization into T-lymphoblasts from Landrace and Piétrain pigs.** Cells were inoculated with six strains of PCV2 particles for 1 h at 37 ℃, followed by IF staining to determine viral binding and internalization. PCV2 particles were stained green, and T-lymphoblasts were stained red. All data were obtained from three pigs of each breed for T-lymphoblasts. Within each replicate, the mean was calculated based on ten randomly selected fields. **A** Confocal microscopy was applied to visualize PCV2 particles bound to and internalized into T-lymphoblasts. Some green virus particles were sticking to the cell membrane, while others were located inside the red cell rings and were regarded as internalized particles (shadowed bars). **B** The percentage of T-lymphoblasts with bound and internalized virus was quantitated (a). The fluorescing area of total bound and internalized virus particles (shadowed bars) per PCV2^+^ cell was quantitated as digital pixels with ImageJ software (b). Data represent the means ± SD of three replicates.

Host cell entry is important to initiate successful virus infection. To investigate whether the viral entry is the main factor causing the PCV2 replication difference between Landrace and Piétrain breeds, a binding and internalization assay was conducted. T-lymphoblasts were incubated with PCV2 particles at 37 °C for 1 h allowing for the examination of virus binding and internalization. Figure 2 illustrates the binding and internalization of six PCV2 strains in two pig breeds. Initially, all PCV2 strains were visually detectable in both Landrace and Piétrain pigs using confocal microscopy. Subsequently, variations in viral binding and internalization were observed among the strains (Figure 2A). Figure 2B illustrates the entry efficiency of PCV2, as determined by the percentage of cells with surface-bound/internalized PCV2 and the count of viral fluorescing pixels (both surface-bound and internalized particles). In Landrace pigs, 31.5% (Stoon-1010), 19.5% (1121), 12.9% (1147), 15.3% (09V448), 74.6% (DE222-13) and 85.4% (19V245) of the T-lymphoblasts were respectively detected with the immunostained green fluorescent PCV2 particles binding to the cell surface or inside the cells, with respective 14.0% (Stoon-1010), 5.7% (1121), 3.0% (1147), 3.2%(09V448), 43.1% (DE222-13) and 57.3% (19V245) of the cells showing internalized viral particles. For Piétrain pigs, percentages of PCV2^+^ cells were generally slightly lower than in Landrace pigs (7.2%, 7.7%, 8.6%, 8.6%, 57.5% and 68.7%), with the following percentages of cells with internalized particles: 2.8%, 2.1%, 1.4%, 2.3%, 42.4% and 48.7%, respectively (Figure 2B, panel a). Fluorescent pixel counts were taken for total PCV2 particles per cell (surface-bound and internalized) and for only internalized viral particles per cell (Figure 2B, panel a). During a one-hour incubation at 37 °C, significant internalization into a substantial number of cells occurred in both breeds, with 38.1–88.8% for Landrace and 53.2–89.0% for Piétrain of bound particles becoming internalized. Detailed data is provided in Additional file 1. Taken together, Landrace and Piétrain pigs exhibited comparable viral binding and internalization levels in T-lymphoblasts. This suggests that PCV2 binding and entry is not the main factor determining the different viral replication levels between the two breeds.

### T-lymphoblasts express CS and decorin (an indication for DS) but not HS

**Figure 3:**
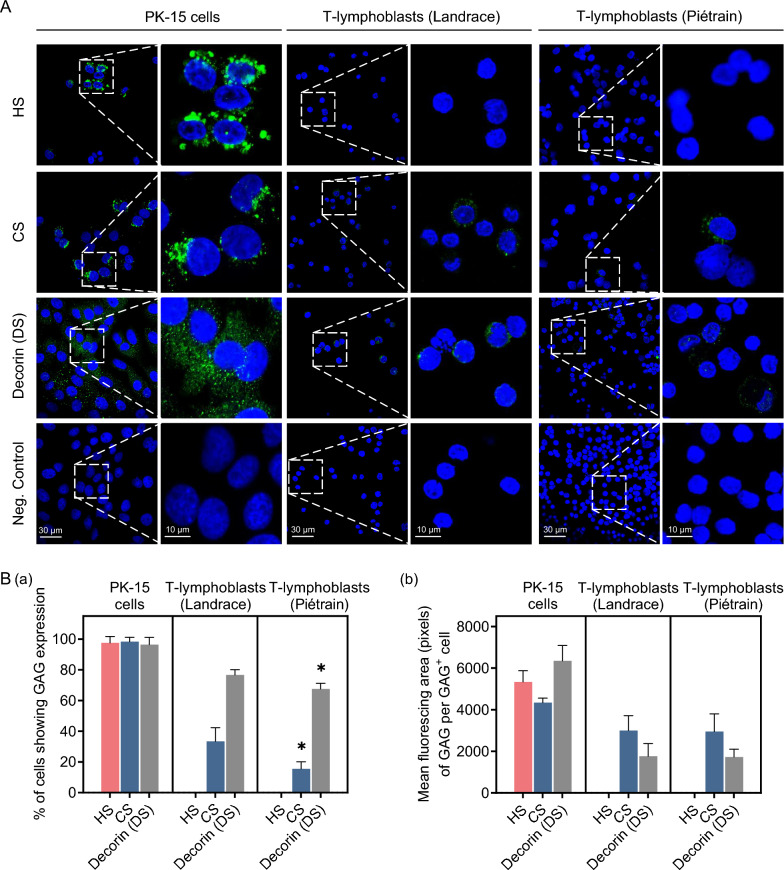
**Expression of HS, CS and decorin (linked with DS chains) on PK-15 cells and T-lymphoblasts from Landrace and Piétrain pigs.** All data were obtained from three different wells for PK-15 cells, and three pigs of each breed for T-lymphoblasts. Within each replicate, the mean was calculated based on ten randomly selected fields. **A** Representative confocal images showing PK-15 cells and T-lymphoblasts stained with specific antibodies against HS, CS and decorin. **B** Quantitation of the percentage of PK-15 cells and T-lymphoblasts expressing HS, CS and decorin (a), and the mean fluorescing area (pixels) of GAG per GAG^+^ cell (b). Data represent the means ± SD of three replicates. Differences between T-lymphoblasts from the two breeds were revealed by the Student’s *t*-test. *P < 0.05.

PCV2 has been identified to utilize HS and DS (also referred to as CS-B) as attachment receptors on porcine kidney 15 (PK-15) cells and the porcine monocytic cell line 3D4/31 [27]. Further, it has been demonstrated that, in contrast to HS, CS is expressed and acts as a receptor for PCV2 on T-lymphoblasts from hybrid Piétrain pigs [20]. To compare the interaction between PCV2 and GAGs in Landrace and Piétrain pigs, we initially determined the expression levels of HS, CS, and DS on the surface of T-lymphoblasts. Specific antibodies targeting HS, CS, and decorin (the protein core associated with DS chains) were used. PK-15 cells, characterized by their elevated expression of HS, CS, and decorin, were included as a positive control in the experiment. In Figure 3B, HS, CS, and decorin (DS) exhibited a strong expression in nearly all PK-15 cells. Consistent with previous findings, HS expression was undetectable on T-lymphoblasts from both breeds. T-lymphoblasts generally exhibited lower expression of CS and decorin (DS) as compared to PK-15 cells. In comparative analysis between the two breeds, Landrace pigs exhibited 33.4% of CS^+^ cells and 76.7% of decorin (DS)^+^ cells, while Piétrain pigs displayed 15.5% of CS^+^ cells and 67.5% of decorin (DS)^+^ cells. Protein expression was also quantified in fluorescent pixels for each GAG^+^ cell. No significant differences were noted in the expression of CS or decorin (DS) between T-lymphoblasts from the two breeds.

### CS and decorin (DS) mediate in part PCV2 binding to T-lymphoblasts

**Figure 4:**
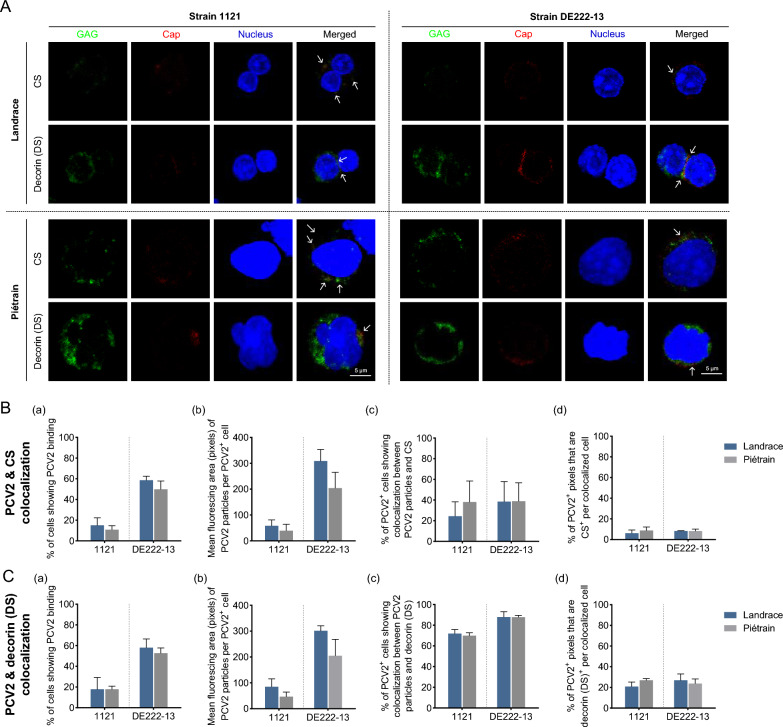
**Co-localization of GAGs [CS, decorin (DS)] and PCV2 particles on T-lymphoblasts of Landrace and Piétrain pigs.** Cells were incubated with PCV2 particles for 1 h at 4℃. A double IF staining was performed to detect GAGs and PCV2 capsid proteins. GAGs were stained in green, and the PCV2 virions were stained in red. Cell nuclei were co-stained in blue. All data were obtained from three pigs of each breed for T-lymphoblasts. Within each replicate, the mean was calculated based on ten randomly selected fields. Visualization of co-localization was performed by confocal microscopy. **A** Representative confocal images of cells with 1121 and DE222-13 particles colocalized with GAG. Co-localization between CS/decorin (DS) and PCV2 within PCV2^+^ T-lymphoblasts was indicated by white arrows. **B**, **C** Co-localization assays between PCV2 particles and CS/decorin (DS). Specifically, the percentage of PCV2^+^ cells was examined (a); fluorescing area of bound PCV2 particles per PCV2^+^ cell was quantitated using ImageJ in pixels (b); the percentage of cells displaying co-localization between PCV2 particles and GAG was assessed (c); for each PCV2-GAG colocalized cell, the proportion of PCV2^+^ pixels that are also GAG expression was determined based on individual fluorescent pixels per cell (d). Data are represented as means ± SD from three replicates.

PCV2a strain 1121 and PCV2d strain DE222-13 are representative strains that enter host cells inefficiently and efficiently, respectively. To examine whether PCV2 binds to T-lymphoblasts through CS and/or decorin (DS), a double IF assay was conducted before virus incubation at 4 °C for 1 h. PCV2 particles were stained with mouse anti-PCV2-Cap monoclonal antibodies, while CS and decorin (DS) were specifically stained with their corresponding anti-CS and anti-decorin antibodies (Figure 4A). The binding of virus particles to the cell surface differed between viral strains; DE222-13 showed a superior binding compared to strain 1121 (Figure 4B and C, panels a, b). In Landrace pigs, the co-localization between PCV2 and CS was observed in 24.4% of PCV2^+^ cells for strain 1121, while the co-localization percentage increased to 38.5% for strain DE222-13. In Piétrain pigs, both strains exhibited a co-localization percentage of 38.0% (Figure 4B, panel c). Further analysis of co-localization per cell (PCV2^+^ pixels that are also GAG-positive) revealed no significant differences in the co-localization ratio between strains or breeds, with values remaining below 10.0% (Figure 4B, panel d). Regarding decorin (DS), strain 1121 and DE222-13 exhibited co-localization with 71.9% and 87.9% of PCV2^+^ cells in Landrace pigs, and 69.8% and 87.8% of PCV2^+^ cells in Piétrain pigs, respectively (Figure 4C, panel c). Again, there were no significant differences in the co-localization ratio between the two strains or between the two breeds, with values ranging from 20.0% to 25.0% per PCV2^+^ cell showing co-localization (PCV2^+^ pixels that are also GAG-positive) (Figure 4C, panel d).

Taken together, viral attachment to T-lymphoblasts is strain-dependent, with certain strains exhibiting a binding advantage. No differences were observed in the co-localization of PCV2 particles with CS and decorin (DS) between strains or breeds, indicative of comparable interactions with these sugar molecules. The PCV2 strains 1121 and DE222-13 only partially relied on binding to these molecules, suggesting that additional target molecules are involved in the viral entry into T-lymphoblasts.

### PCV2 internalization into T-lymphoblasts is partially mediated by phosphacan

**Figure 5:**
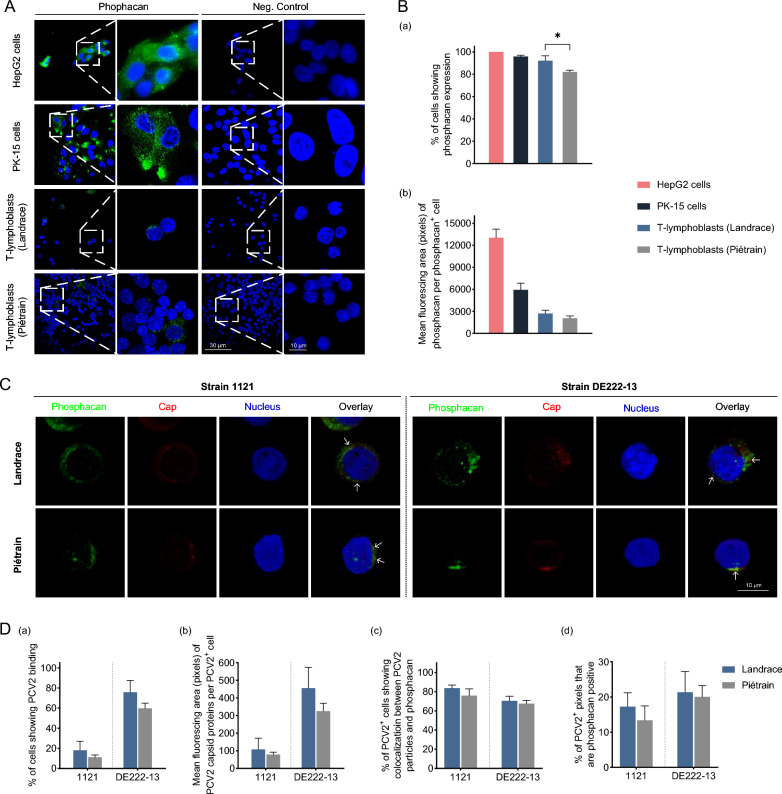
**Phosphacan expression on T-lymphoblasts and its co-localization with PCV2 virions.** All data were obtained from three pigs of each breed, and within each replicate, the mean was calculated based on ten randomly selected fields. **A** Visualization of phosphacan expression on T-lymphoblasts from Landrace and Piétrain pigs. **B** The percentage of T-lymphoblasts expressing phosphacan (a) and the mean fluorescing area of phosphacan per phosphacan^+^ cell (b) were quantitated. **C** Confocal microscopy was used to visualize the co-localization between phosphacan and PCV2. Phosphacan and PCV2 virions were stained green and red, respectively. **D** Co-localization assays between phosphacan and PCV2 particles were conducted, including examination of the percentage of PCV2^+^ cells (a), quantification of the fluorescing area of attached PCV2 particles per PCV2^+^ cell using ImageJ (b), determination of the percentage of cells displaying co-localization between phosphacan and PCV2 particles (c), and evaluation of the proportion of PCV2^+^ pixels that are also exhibited phosphacan expression for each PCV2-phosphacan colocalized cell based on individual fluorescent pixels per cell (d). Data are represented as means ± SD from three replicates. Statistical differences were determined by the Student’s *t*-test. *P < 0.05.

Phosphacan, a glycoprotein found widely on the cell surface, is identified as a potential transmembrane molecule that mediates the uptake of PCV2 particles by monocytes. This is attributed to its ability to interact with three positively charged bands (a three-wings-windmill-shaped pattern) of PCV2 capsids through its multiple CS chains [21]. To elucidate the role of phosphacan in the PCV2 infection of T-lymphoblasts, we initially evaluated the presence of phosphacan on the surface of T-lymphoblasts (Figure 5A). Similar to HepG2 cells, which were used as a positive control, phosphacan expression was observed on the majority of T-lymphoblasts, confirming its presence in these cells. However, the percentage of phosphacan^+^ cells was lower in Piétrain pigs (82.12%) compared to Landrace pigs (92.07%). In terms of phosphacan expression per phosphacan^+^ cell, it was found to be relatively low in both pig breeds, with only 20.8% (Landrace) and 15.9% (Piétrain) of the expression level detected in HepG2 cells (Figure 5B).

Afterwards, the co-localization of PCV2 particles with phosphacan was assessed through a double IF staining using specific anti-PTPRZ1 and anti-PCV2-Cap antibodies (Figure 5C). The percentage of co-localization among PCV2-positive cells showed no significant difference, registering around 80% for strain 1121 and 70% for DE222-13. The co-localization ratios between the strains and breeds also did not differ significantly, with values ranging from 13.4% to 21.4% per PCV2^+^ cell showing co-localization (PCV2^+^ pixels that are also GAG-positive) (Figure 5D).

### Inhibition of endo-lysosomal acidification diminishes PCV2 infection in T-lymphoblasts from Landrace but not Piétrain pigs

Most viruses, whether enveloped or not, utilize endocytic entry mechanisms during host cell infection. A crucial step in this process is the acidification of the endosome-lysosome system, which triggers virus penetration into the host cell. PCV2 infection in T-lymphoblasts also needs a low-pH stage, emphasizing the importance of endo-lysosomal acidification in the viral entry process. To determine the potential association between intracellular acidification and the different susceptibility of Landrace and Piétrain pigs to PCV2 infection, CQ was used to block the pH drop within endosomal compartments. The effect of PCV2 infection in the two breeds was subsequently examined.

**Figure 6:**
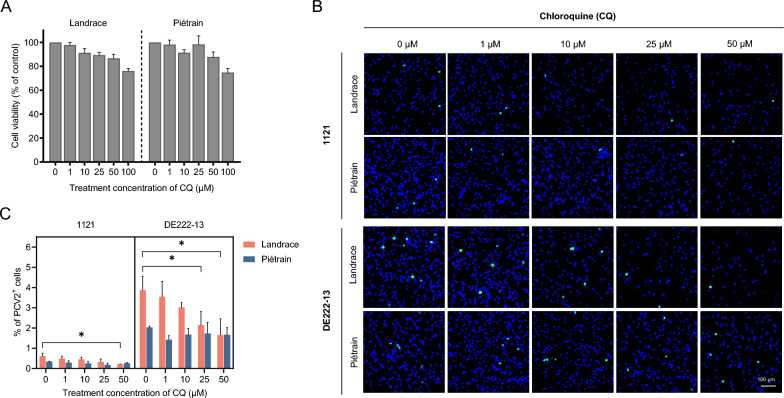
**Effect of inhibition of endo-lysosomal acidification on PCV2 infection of T-lymphoblasts in Landrace and Piétrain pigs.** **A** Cell viability of T-lymphoblasts was detected by a CCK-8 assay kit under CQ treatment at different concentrations. **B** Visualization of PCV2 infection in T-lymphoblasts after CQ treatment. **C** Percentage of cells displaying PCV2 infection. Cells were pre-treated with CQ (0, 1, 10, 25 and 50 μM) prior to PCV2 incubation, and the cells were cultured in the presence of CQ until 24 h. The percentage of PCV2^+^ cells (stained in green) was quantitated. All data were obtained from three pigs of each breed, and within each replicate, the mean was calculated based on ten randomly selected fields. Data represent means ± SD of triplicated assays. Statistical differences were determined by the Student’s *t*-test. *P < 0.05.

Pre-treatment of T-lymphoblasts with varying concentrations of CQ revealed a dose-dependent effect on PCV2 infection in Landrace pigs, resulting in a significant reduction compared to untreated cells. A maximum reduction of 57.1–65.2% in PCV2 infection was observed at a concentration of 50 μM CQ (Figure 6). Intriguingly, in Piétrain pigs, PCV2 infection did not display a significant decline following CQ treatment for both PCV2 strains. These findings collectively demonstrate that PCV2 infection of T-lymphoblasts in Landrace pigs relies in part on a low-pH environment, while this low-pH-dependent mechanism is not necessary in Piétrain pigs.

### Serine proteases are essential for PCV2 infection in T-lymphoblasts from both Landrace and Piétrain pigs

**Figure 7:**
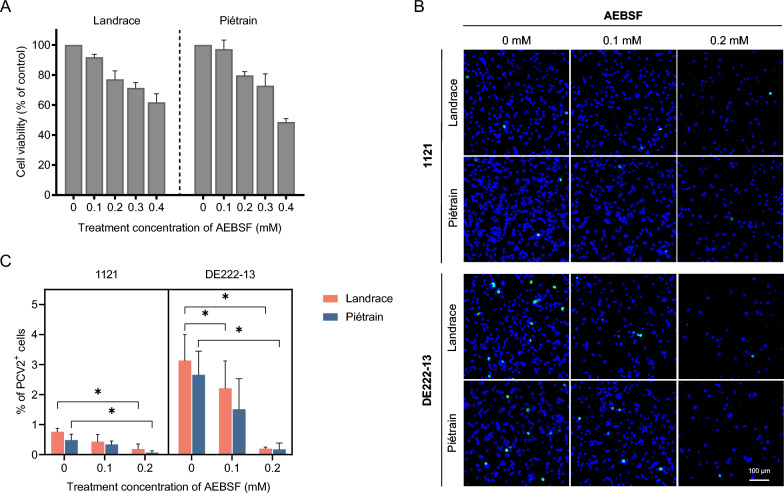
**Effect of inhibition of serine proteinases on PCV2 infection of T-lymphoblasts in Landrace and Piétrain pigs.** **A** Cell viability of T-lymphoblasts was detected by a CCK-8 assay kit under AEBSF treatment at different concentrations. **B** Visualization of PCV2 infection in T-lymphoblasts after AEBSF treatment. **C** Percentage of cells displaying PCV2 infection. Cells were pre-treated with AEBSF (0, 0.1 and 0.2 mM) prior to PCV2 incubation, and the cells were cultured in the presence of AEBSF for 16 h and further cultured in fresh medium without AEBSF until 24 h. The percentage of PCV2^+^ cells (stained in green) was quantitated. All data were obtained from three pigs of each breed, and within each replicate, the mean was calculated based on ten randomly selected fields. Data represent means ± SD of triplicated assays. Statistical differences were determined by the Student’s *t*-test. *P < 0.05.

Viral disassembly within the endosome-lysosome system is a complex process involving the concerted action of various cellular proteases, including serine, aspartic, cysteine, and metalloproteases [28]. Serine proteases, among these protease families, have been identified as key players in PCV2 disassembly across various cell types, including PK-15 cells, 3D4/31 monocytic cells, and T-lymphoblasts [20, 29]. To study the potential influence of serine proteases in differential susceptibility to PCV2 infection in T-lymphoblasts from Landrace and Piétrain pigs, cells were pretreated with the serine protease inhibitor AEBSF at various concentrations prior to PCV2 inoculation. The inhibition of serine proteases led to a dramatic reduction in PCV2 infection in both Landrace and Piétrain pigs. Treatment with AEBSF at a concentration of 0.2 mM significantly blocked PCV2 infection with 75.2–93.7% in Landrace pigs and with 86.0–93.4% in Piétrain pigs, compared to the non-treated group (Figure 7). Therefore, one can conclude that serine proteases are essential for PCV2 infection in T-lymphoblasts from both Landrace and Piétrain pigs.

### Cathepsin G (CatG) plays a limited role in PCV2 infection of T-lymphoblasts

**Figure 8:**
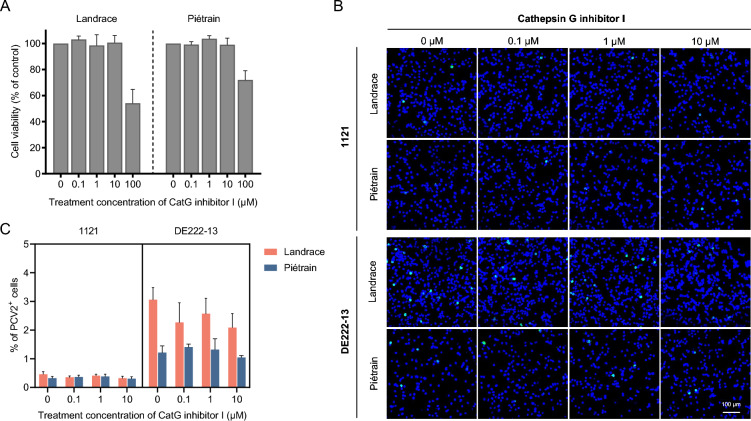
**Effect of inhibition of Cathepsin G (CatG) on PCV2 infection of T-lymphoblasts in Landrace and Piétrain pigs.** **A** Cell viability of T-lymphoblasts was detected by a CCK-8 assay kit under CatG inhibitor I treatment at different concentrations. **B** Visualization of PCV2 infection in T-lymphoblasts after CatG inhibitor I treatment. **C** Percentage of cells displaying PCV2 infection. Cells were pre-treated with CatG inhibitor I (0, 0.1, 1 and 10 μM) prior to PCV2 incubation, and the cells were cultured in the presence of AEBSF until 24 h. The percentage of PCV2^+^ cells (stained in green) was quantified. All data were obtained from three pigs of each breed, and within each replicate, the mean was calculated based on ten randomly selected fields. Data represent means ± SD of triplicated assays. Statistical differences were determined by the Student’s *t*-test.

CatG, a serine protease that operates at a neutral pH, is predominantly located within the endo-lysosomal system and is pH-independent [30, 31]. Considering the observed pH-drop-independence of PCV2 infection in Piétrain T-lymphoblasts, we hypothesized that CatG might be responsible for the unaltered PCV2 infection in these cells when endo-lysosomal acidification is inhibited. To investigate CatG’s potential role in PCV2 infection, this study employed a CatG inhibitor. As shown in Figure 8, treatment with the CatG inhibitor I resulted in a non-significant reduction of PCV2 infection in both Landrace and Piétrain pigs. The maximum decrease was 29.7% for strain 1121 and 31.8% for strain DE222-13 at a concentration of 10 μM in Landrace pigs, and 4.9% for strain 1121 and 13.9% for strain DE222-13 at the same concentration in Piétrain pigs. Thus, CatG seems to play a limited role in PCV2 infection of T-lymphoblasts and is not the principal factor in the neutral pH-dependent PCV2 infection in Piétrain T-lymphoblasts. Other proteases should be considered.

### Increased susceptibility to PCV2 in Landrace T-lymphoblasts is likely attributed to viral escape occurring within late endosomes

PCV2 enters host cells primarily through clathrin-mediated endocytosis and subsequently undergoes trafficking along the endosome-lysosome pathway, where it faces the fate of either escape or degradation [20, 32, 33]. This observation gave rise to the hypothesis that differences in the trafficking kinetics of PCV2 virions in the endo-lysosomal pathway between Landrace and Piétrain pigs may account for the contrasting viral susceptibility observed in these breeds.

#### Kinetics of viral degradation within lysosomes vary between Landrace and Piétrain pigs

**Figure 9:**
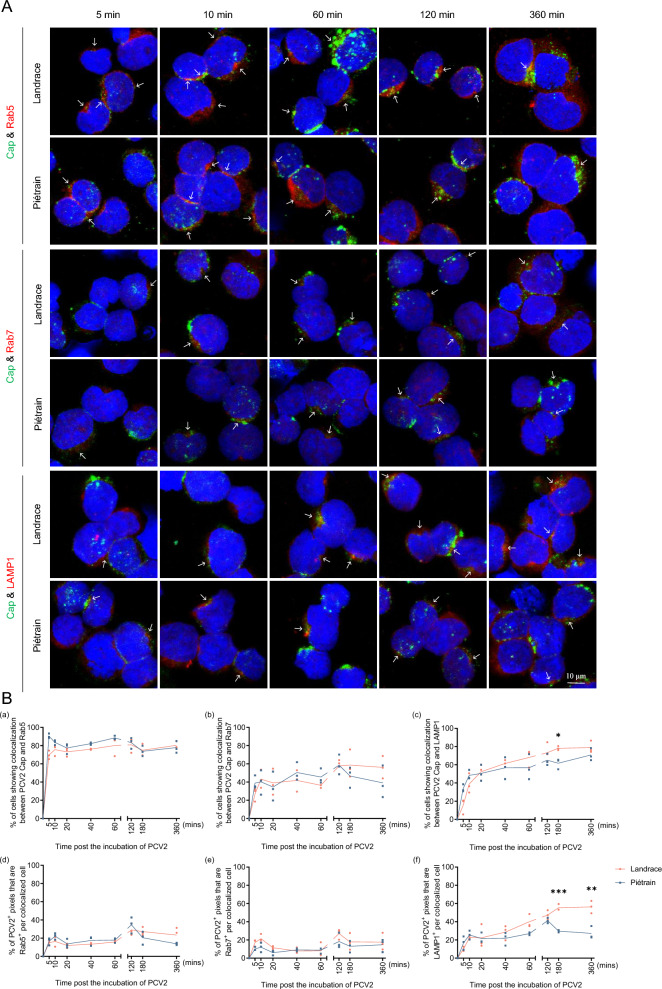
**Co-localization analysis of PCV2 intracellular trafficking.** Confocal microscopy was used to visualize the merged time-course analysis of representative cells showing co-localization between PCV2 capsid proteins and early endosome markers (Rab5), late endosome markers (Rab7), and lysosome markers (LAMP1). Virions were stained in green, and endo-lysosomal markers were stained in red. Nuclear morphology was visualized with Hoechst 33342 (blue) counterstaining. **A** IF staining was conducted to visualize PCV2 co-localization with Rab5, Rab7, and LAMP1, respectively. **B** The percentage of cells displaying co-localization between Cap and Rab5/Rab7/LAMP1 (a–c) and the co-localization ratio indicating the proportion of PCV2^+^ pixels that also exhibited Rab5/Rab7/LAMP1 expression for each colocalized cell based on individual fluorescent pixels per cell (d–f) were quantified. Statistical differences were determined by the Student’s *t*-test. *P < 0.05, *** P < 0.001.

To test this hypothesis, a co-localization assay was performed for on the one hand the PCV2 (strain DE222-13) Cap protein and on the other hand early endosomes (EEs), late endosomes (LEs), and lysosomes, using specific antibodies against Rab5, Rab7 and LAMP1 respectively. The PCV2 Cap protein was immunostained green, and the cell organelle markers were stained red. Co-localization was identified through the merging of green fluorescence from the viral protein with the red fluorescence of the corresponding cell organelles. The green fluorescent spots at the cell periphery indicate viruses that are attached to the cell surface but not yet internalized (Figure 9A).

In Figure 9B, the first column depicts the percentage of cells demonstrating the presence of both viral particles and EEs (panel a), LEs (panel b), and lysosomes (panel c) on the same cell. The second column illustrates the percentage of co-localization fluorescence pixels between PCV2 Cap proteins and EEs (panel d), LEs (panel e), and lysosomes (panel f) per colocalized cell, relative to the mean Cap fluorescence pixels per PCV2^+^ cell (hereinafter referred to as the co-localization ratio). Generally, the trafficking kinetics of PCV2 in EEs and LEs of T-lymphoblasts from Landrace and Piétrain pigs showed little difference, with significant differences primarily observed during the entry into lysosomes. After internalization into cells, the virus rapidly reaches EEs, as shown in Figure 9B, panels a–c, where over 69.6% of PCV2^+^ cells exhibited co-localization with Rab5 at 5 min. As the EEs mature into LEs, the virus subsequently enters LEs, with more than 40.2% of viral capsid proteins colocalizing with LEs at 10 min. Subsequently, more and more viruses enter lysosomes until the end of the experiment (360 min). In Landrace pigs, around 73.3% of cells showed co-localization between PCV2 capsid proteins and LAMP1 at 120 min, while it was approximately 65.0% in Piétrain pigs. However, some viral proteins were observed to still colocalize with Rab7^+^ endosomes, signifying a slow and sustained exit of the virus from LEs.

Regarding the co-localization ratio (Figure 9B, panels d–f), no significant difference in Cap-EE/LE co-localization was observed over time in either pig breed. However, in lysosomes, the co-localization ratio between PCV2 Cap and LAMP1 in Landrace continued to increase until the end of the experiment, while this co-localization in Piétrain remained consistent with the trends observed in EEs and LEs, reaching the maximum difference at 360 min (56.3% in Landrace pigs, 27.2% in Piétrain pigs). This observation suggests that PCV2 Cap proteins continuously accumulate in Landrace T-lymphoblast lysosomes, whereas they degrade more efficiently in Piétrain T-lymphoblast lysosomes. In conclusion, the results demonstrate that PCV2 traffics along the endo-lysosomal pathway after internalization into T-lymphoblasts in both Landrace and Piétrain pigs, with differing degradation kinetics within lysosomes.

#### Viral escape may occur in late endosomes in Landrace T-lymphoblasts

**Figure 10:**
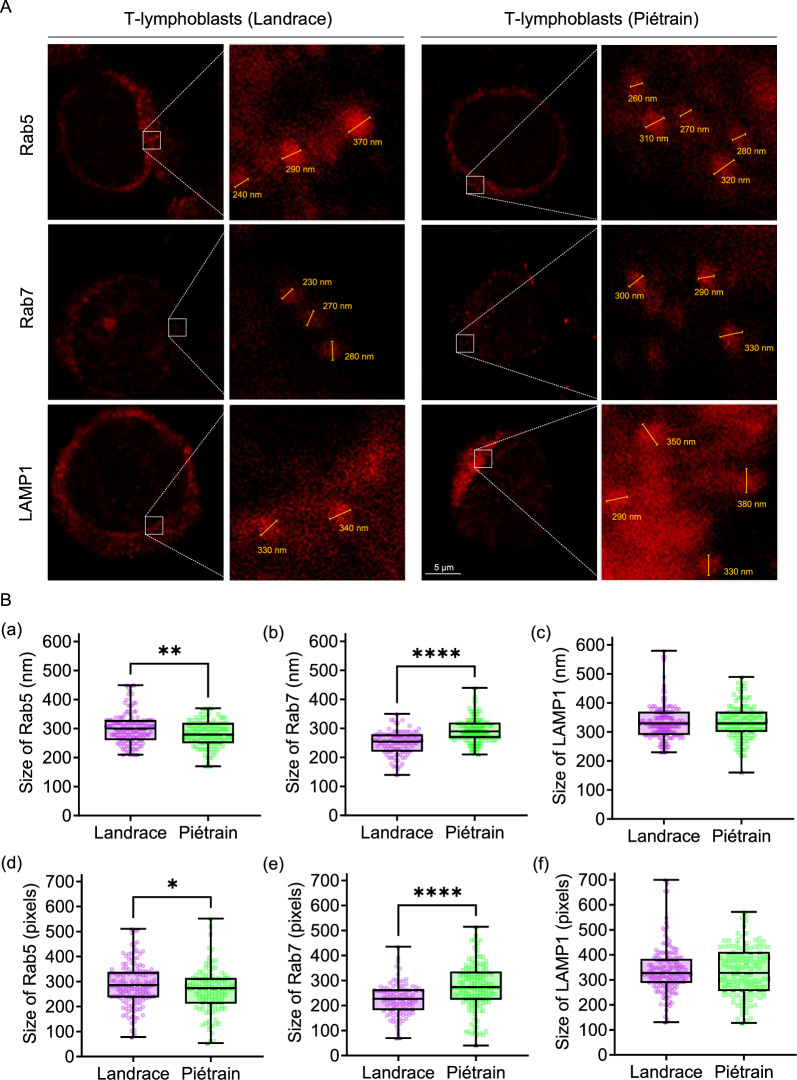
**Organelle size analysis during PCV2 intracellular trafficking.** Early endosomes (EEs), late endosomes (LEs) and lysosomes were immunostained with specific antibodies and followed by size measurement with confocal microscopy. **A** Representative confocal images displaying the expression of Rab5, Rab7 and LAMP1 indicating EEs, LEs, and lysosomes, respectively. The size of these organelles was analyzed using Leica Application Suite X software and labelled in yellow. **B** Quantification of the size, including the diameter (a–c) and the pixels (d–f) of Rab5 (5 min), Rab7 (10 min) and LAMP1 (120 min) at specific time points. The number of counted organelles is more than 100. The pixel size of the fluorescence images corresponds to 24.3 nm. Data are represented as means ± SD from three replicates (three pigs from each breed). Statistical differences were determined by the Student’s *t*-test post-Welch’s correction. *P < 0.05, **P < 0.01, **** P < 0.0001.

To probe deeper into the virus’s trafficking within the endo-lysosomal system, we measured and compared the sizes of EEs, LEs, and lysosomes between Landrace and Piétrain pigs. The sizes were determined by the diameter (nm) and fluorescence area (pixel) of their respective markers, Rab5, Rab7, and LAMP1. As shown in Figure 10B, the average diameter and fluorescence area of EEs in Landrace T-lymphoblasts were larger, while the size of LEs was smaller compared to those in Piétrain pigs. Lysosome sizes did not significantly differ between the two breeds. The average diameter of EEs in Landrace T-lymphoblasts was 303 nm, with an average fluorescence area of 290 pixels, compared to 282 nm with an average fluorescence area of 266 pixels in Piétrain T-lymphoblasts. The variation in EE size may be associated with the virus entry-level, consistent with the observation in Figure 2 of a slightly higher level of virus entry in these cells compared to Piétrain T-lymphoblasts. The statistical significance observed in Figure 10B, but not in Figure 2B, might stem from the larger data set utilized here, encompassing measurements from over 100 fluorescence spots, thereby enhancing the sensitivity of the comparison.

Regarding LEs, the average diameter in Landrace pigs was 251 nm, and the average fluorescence area was 227 pixels, whereas, in Piétrain pigs, it was 291 nm with an average fluorescence area of 276 pixels. The reduced size of LEs in Landrace pigs, smaller even than their EEs, implies that, upon protease cleavage of the capsid, more expanding viral genome-rings may escape from LEs, leading to infection.

Concerning the lysosome size, the two pig breeds showed no significant difference. This might result from lysosomes serving as cellular waste stations, where diverse cellular and non-cellular debris accumulate post-virus internalization. For lysosomal proteases, it takes time to fully process these waste materials. As for the viruses, they might leave only empty or incomplete capsid shells upon arrival at lysosomes.

## Discussion

PCV2 is one of the most economically important pig pathogens affecting the global pig industry. Understanding the mechanisms of viral entry, disassembly, and replication in natural target cells is crucial for developing therapeutic interventions against PCV2 infection and screening for resistant pigs. PCV2 primarily targets monocytes and lymphoblasts. Monocytes can take up PCV2 virions but do not support efficient virus replication [11, 33, 34]. In contrast, lymphoblasts exhibit a high susceptibility to PCV2 infection. A greater number of these susceptible cells by all conditions that cause lymphoblastogenesis (e.g., co-infections, vaccinations) contributes to an accelerated and more pronounced primary replication of PCV2 within the host [11–13].

Previous studies have indicated a breed preference in PCVADs, with Landrace pigs exhibiting more severe clinical symptoms than Piétrain pigs. This suggests that Piétrain pigs are more resistant to PCV2 infection [18, 19]. Our present results suggest that differences in viral disassembly and release from disrupted endosomes may explain the contrasting PCV2 susceptibility in T-lymphoblasts of Landrace and Piétrain pigs.

PCV2 replication in T-lymphoblasts is breed and strain-dependent, replicating more efficiently in Landrace pigs compared to Piétrain pigs, a finding consistent with clinical observations. We examined six typical PCV2 strains including PCV2a, PCV2b and PCV2d, which were or are currently prevalent worldwide. The study demonstrates that viruses originating from PCVAD cases, characterized by high viral loads in lymph nodes (indicative for PMWS), replicate very efficiently in T-lymphoblasts. Especially, the most recent PCV2d-2 strains DE222-13 and 19V245 demonstrated higher replication levels in T-lymphoblasts compared to PCV2d-1 strain 09V448 and PCV2a strain Stoon-1010, while PCV2a strain 1121 and PCV2b strain 1147 exhibited the weakest replication (Figure 1). Thus, the aforementioned findings suggest a correlation between, on the one hand, PMWS-associated strains, which cause a high replication in lymphoblasts leading to a massive lysis of these cells and general dysregulation of the immune system and, on the other hand, susceptible pig breeds (such as Landrace). The higher PCV2 replication in Landrace pigs is not the only factor leading to high virus titers in affected animals. In a previous paper from our group, it was demonstrated that PCV2 virion uptake and destruction is less efficient in Landrace compared to Piétrain pigs [21]. We believe that PCV2 evolves in a direction that is in favor of the virus: a better replication in lymphoblasts and a worse elimination by monocytes. This fits in the Darwinian law of the fittest. Of course, this evolution occurs in pig breeds that are on the rise in the world pig production. This is a very dangerous evolution, especially, when pigs are in a continuous lymphoblastogenesis stage due to overlapping chains of infections and vaccination. A limit is reached at this moment. This high blastogenesis stage is also giving rise to other small DNA viruses, that need cellular polymerases to replicate, such as other circoviruses (PCV3 and PCV4), a large number of parvoviruses, papillomaviruses and polyomaviruses [35–39]. It is important that in the future, the number of infections that pigs have to deal with will reduce. Otherwise, we enter in a vicious circle of more viruses that replicate in lymphoblasts and stronger blastogenesis levels.

In this evolution context, the Cap is mutating to bind and internalize better in lymphoblasts [24]. As we illustrated in our previous study in PCV2 uptake by monocytes, amino acid variations were observed in the six PCV2 strains, with certain charged amino acids on the capsid surface potentially influencing initial host cell binding. Capsid 3D analysis of capsid structures showed that, distinct from strains 1121 (PCV2a), 1147 (PCV2b) and 09V448 (PCV2d-1), PMWS-associated strains Stoon-1010 (PCV2a), DE222-13 (PCV2d-2) and 19V245 (PCV2d-2) displayed three complete positively charged bands on a threefold axis, generating a counterclockwise three-wings-windmill pattern. This facilitates the binding between the PCV2 virion and phosphacan via the electrostatic interaction between the three-wings-windmill-like positively charged bands and any three negative GAG chains on phosphacan, leading to viral particles accumulation on the cell surface [21].

Successful viral infection commences with efficient binding of the virus to the cell surface, followed by its subsequent internalization. In this study, we compared the viral binding and internalization in lymphoblasts of Landrace and Piétrain pigs. PCV2 exhibits a slightly higher attachment to T-lymphoblasts of Landrace pigs than Piétrain pigs. However, the viral internalization ratio does not significantly differ between the two breeds, suggesting that viral attachment and internalization are not the main reasons for the different susceptibility of PCV2 in different host breeds. To further assess if the difference in PCV2 susceptibility in T-lymphoblasts of Landrace and Piétrain pigs can be explained by viral binding and internalization, we investigated the role of GAGs. GAGs are polysaccharides composed of repeating disaccharide units. They are heterogeneous, negatively charged, unbranched, and linear in structure. Typically, they covalently attach to various core proteins to form proteoglycans (PGs). They act as essential cell surface molecules, mediating virus binding through electrostatic interactions. GAGs are categorized into HS, CS, DS, keratan sulfate (KS), heparin and hyaluronan (HA) [40, 41]. Our study focused on the interaction between PCV2 virions and the first three GAGs, as the other types are not expressed on T-lymphoblasts [20]. Unlike the strong expression of HS and CS in epithelial cell lines PK-15, ST, and SK [27], T-lymphoblasts exhibited no expression of HS, with only a restricted portion of cells showing limited expression of CS and DS. Leukocytes are known to primarily express GAGs of the CS type. This finding is consistent with our observation that little or no cell surface HS is detectable on human mitogen-activated T cells [42, 43].

To evaluate the roles of CS and DS in PCV2 infection in T-lymphoblasts, we investigated the co-localization of GAG and viral particles. Although CS and DS were expressed in approximately 25–38% and 70–88% of PCV2-bound cells respectively, the co-localization ratio of fluorescence pixels per colocalized cell was rather low, with CS at around 10% and DS at approximately 20%. This would mean that about 70% of viral attachment is mediated by other molecules. One should be careful with a too strong conclusion here as it is possible that the GAGs were present at low levels that could not be identified by immunofluorescence. Consistent with our findings, previous studies have demonstrated that PCV2 can also infect GAG-deficient CHO cells [27]. The binding of strongly-replicating DE222-13 to CS and DS was similar to that of the low-replicating 1121, indicating that the more efficient binding of DE222-13 particles to T-lymphoblasts compared to 1121 was not mainly caused by GAGs. These findings suggest that, in addition to the two identified GAG receptors responsible for PCV2 attachment, there are other yet-unidentified cell surface molecules involved in viral attachment, and they may exhibit a preference for specific strains. The differences in HS and CS expression in T-lymphoblasts and PK-15 cells partially account for the distinct characteristics of PCV2 attachment to these two cell types. Overall, further exploration of PCV2 receptors on host cells is required.

In T-lymphoblasts, phosphacan expression suggests its potential involvement in PCV2 internalization, similar to primary monocytes [21]. Although phosphacan expression per cell is relatively low, it is widely present on T-lymphoblasts of both Landrace (92.1%) and Piétrain (82.1%) pigs. The variation in phosphacan^+^ cell percentages between the two pig breeds may reflect breed-specific differences in phosphacan expression or regulation. Over 70% of PCV2^+^ cells also express phosphacan, however the average co-localization ratio per cell is below 20%. This suggests that phosphacan plays a partial role in PCV2 internalization in T-lymphoblasts, with other factors or molecules likely contributing to the entry process. Therefore, considering the findings of GAGs as attachment molecules and phosphacan as an internalization mediator, it is essential to search for other cell surface receptors for PCV2 infection in T-lymphoblasts. The slight difference in viral entry between the two pig breeds seems insufficient to explain the differences in PCV2 pathogenesis. Therefore, we investigated subsequent events following viral entry into cells.

PCV2 enters T-lymphoblasts via clathrin-mediated endocytosis [20]. After internalization, viral particles undergo uncoating, releasing the viral genome for replication at suitable sites. The uncoating process is host cell-dependent, similar to internalization. In 3D4/31 monocytic cells, PCV2 requires an acidic environment for uncoating, while in epithelial cell lines, such as PK-15, SK, and ST, preventing the pH decrease boosts PCV2 replication [29, 32]. Interestingly, we observed that a decreased intracellular pH promotes PCV2 replication in Landrace T-lymphoblasts but has minimal effect on Piétrain T-lymphoblasts. This suggests a pH-dependent and independent infection in Landrace T-lymphoblasts, and a pH-independent infection in Piétrain T-lymphoblasts. Furthermore, we demonstrated that PCV2 capsid disassembly, is mediated by serine proteases. At 0.2 mM AEBSF, PCV2 infection rates decreased by over 75% in both pig breeds. Considering the CQ inhibition findings, serine proteases, with an activity at acidic and neutral pH, are active during viral disassembly. In Piétrain pigs, neutral pH serine proteases are active in the viral disassembly. Landrace pigs use, on top of this, acidic pH serine proteases which results in an enhanced viral infection. We explored further if CatG, a major serine protease with neutral pH activity, may be one of the neutral pH-dependent proteases that are active during a PCV2 infection in Piétrain pigs. Inhibition experiments revealed that CatG is not the primary proteolytic enzyme responsible for PCV2 disassembly at a neutral pH. Further research is needed to identify the serine proteases with activity at different pH levels in the endosomes/lysosomes, affecting PCV2 infection in T-lymphoblasts from various breeds, including Cathepsin A, elastase, protease 3, and granzymes A/B, among others [44–46].

Endocytosis is a sophisticated and dynamic cellular process that encompasses the recycling, trafficking, maturation, and fusion of endocytic vesicles [47]. After formation, these vesicles are transported to EEs with a slightly acidic pH. Subsequently, EEs mature into LEs or multivesicular bodies, where the pH gradually decreases. Eventually, LEs fuse with lysosomes, which have an even lower pH, leading to the degradation of the internalized cargo [48–50]. Viruses encounter challenges during endocytosis, as they need to escape the endosomal compartment to prevent recycling back to the extracellular space or degradation in the acidic lysosomal environment [51–53]. Enveloped viruses employ complex molecular mechanisms to facilitate the fusion of the viral envelope with the endosomal membrane, enabling them to bypass the endosomal pathway and escape. In contrast, non-enveloped viruses have developed diverse strategies to disassemble and enter the cytoplasm of the host cell [54]. PCV2 is a non-enveloped virus that does not disassemble by fusing with the endosomal membrane. During infection, viral particles experience conformational changes to respond to various external stimuli [55–57], such as binding to cell receptors [58, 59], proteolysis [60, 61], changes in bivalent cation concentrations [62, 63], or endosomal acidification [60, 64, 65]. Moreover, membrane-modifying proteins physically penetrate the endosomal membrane [53], causing endosome membrane distortion and disruption. As a result, the virus genome is released into the cytoplasm and subsequently delivered into the nucleus, initiating the virus replication cycle and establishing infection [53, 66, 67]. As the PCV2 genome is a ring that is folded many times to fit into a capsid, it may, upon cleavage of the capsid expand in an explosive way and destroy the endosomal membrane.

To understand how PCV2 particles are transported in the endo-lysosomal pathway of T-lymphoblasts from two pig breeds, we investigated the co-localization of PCV2 Cap proteins with Rab5 (EEs), Rab7 (LEs) and LAMP1 (lysosomes), respectively, which serve as key regulators and markers in this pathway. Our findings revealed a similar localization ratio of Cap proteins in EEs and LEs of both Landrace and Piétrain pigs. However, we observed more Cap co-localization in the lysosomes of Landrace pig. These proteins are likely empty or remnant viral capsids, and the viral genome must escape as early as possible to avoid degradation in the acidic lysosomes [68]. Not all viruses in this study showed co-localization with endo-lysosomal markers. It is possible that it concerns viral capsids that upon genome-ring release and lysis of the endosome, arrived in the cytosol. Otherwise, PCV2 may be recycled back to the cell surface or transported to unmarked organelles/compartments, such as recycling endosomes, the trans-Golgi network (TGN), the endoplasmic reticulum (ER), or amphisomes [69–72]. Further research is needed to explore this. In addition, the proteolytic activity in the lysosomes of Piétrain pigs appears to be stronger than in Landrace pigs, possibly explaining the low accumulation of Cap proteins in the lysosomes. Next, we investigated the sizes of EEs, LEs, and lysosomes at representative time points in order to see if size matters in the context of the escape of the viral genome-ring from the endosomes. When endosomes are small, then it is expected that, upon cleavage of the capsid by proteases, the genome-ring with a theoretical diameter of 191 nm escapes from the capsid and explosively expands leading to the disruption of the endosome [73]. When endosomes are bigger, it is possible that the genome remains inside the endosome and is transported towards the lysosomes for further degradation. After internalization, virions typically enter EEs within 5–10 min [74]. Cargo is retained in EEs for only a few minutes, during which the EEs gradually acidify and mature into LEs [75]. Our findings demonstrated a notable disparity in the size of LEs between Landrace and Piétrain pigs at 10 min after virus internalization, with Landrace pigs exhibiting smaller LEs. (Figure 10B). As the pH is at that moment low, low pH proteases may cleave the capsid and release the PCV2 genome-ring; the ring will explosively expand and destroy the smallest LEs. As Landrace pigs have the smallest LEs and possess low pH proteases that may cleave the capsid, it is logical that PCV2 replicates much better in their T-lymphoblasts compared to Piétrain pigs.

**Figure 11:**
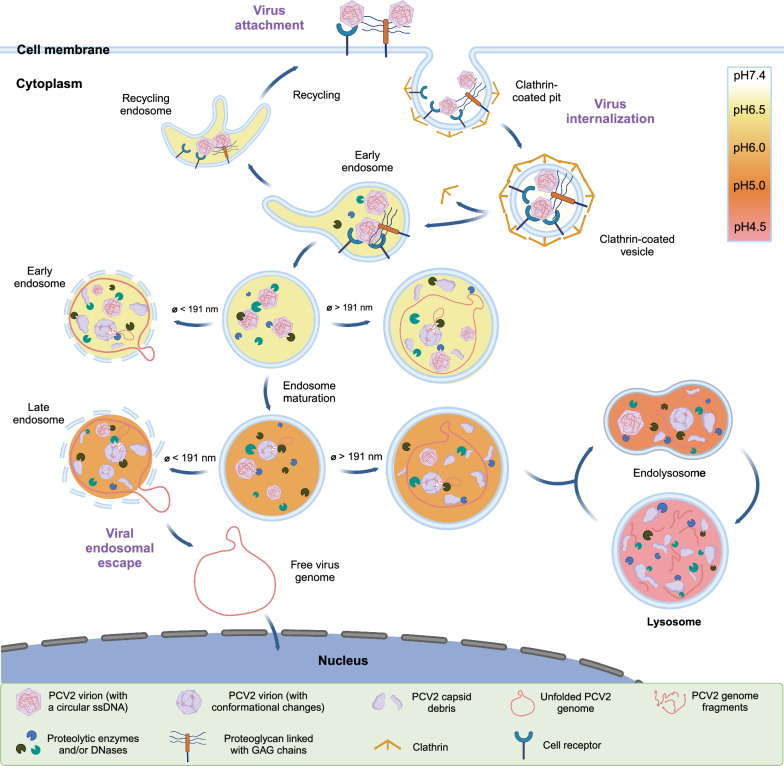
**PCV2 virion trafficking in the endo-lysosomal network.** The process of PCV2 infection begins with the virion attaching to GAGs or specific cell receptors. Viral particles gather on the surface of T-lymphoblasts. The signaling pathway of clathrin-mediated endocytosis is then activated, leading to the formation of clathrin-coated vesicles (CCVs). The vesicle delivers its viral content to the early endosome (EE), where sorting determines whether the vesicle is recycled to the cell surface. The EE containing the virus subsequently undergoes acidification and matures into the late endosome (LE). Virions transfer their genetic materials from the endosomal compartment to the cytoplasm through membrane permeabilization or lysis. Lysis typically occurs when the viral capsid induces a rupture in the endosomal membrane. This process is triggered by conformational changes in the capsid protein, resulting from binding to cell receptors, proteolysis, endosomal acidic pH, or changes in divalent cation concentrations. The outcome is either the release of the viral genome into the cytosol, especially when the endosome is very small and the expanding viral genome-ring destroys it or the residence of the viral genome in the endosome, especially when the endosome is big, and the viral genome cannot escape. The LE then fuses with the lysosome to form an endolysosome, within which the accompanying viral content is degraded until the remaining cargoes are either transported to lysosomes for final degradation or to other possible organelles. Relatively little is known about the other viral locations and the mechanistic interactions between PCV2 virions and the endo-lysosomal network. This figure is created with BioRender.

Figure 11 displays a model that illustrates PCV2 trafficking along the endo-lysosomal pathway, elucidating the fate of virions in T-lymphoblasts and shedding light on viral traffic, endosomal escape, and breed-specific differences in PCV2 infection. Future work should be done on PCV2 cellular receptors, endosomal proteases and viral escape mechanisms within the endo-lysosomal network.

In summary, this study demonstrated a better PCV2 replication in T-lymphoblasts of Landrace pigs (highly susceptible) than Piétrain pigs (low susceptible) and a different replication efficiency among strains. Mechanistically, we demonstrated that: (i) viral binding and internalization are not responsible for this breed difference; (ii) GAGs and phosphacan partially mediate viral entry; (iii) PCV2 utilizes serine proteases to disassemble and infect lymphoblasts at a neutral pH for both breeds but also at a low pH for Landrace only; (iv) viral escape from LEs may explain the higher susceptibility in Landrace pigs. These findings provide novel evidence for the host genetics’ significant role in PCV2 infection, illuminating the underlying mechanisms involved in the complex pathogenesis of PCVADs. This understanding is expected to facilitate the development of therapeutic interventions and resistant pigs.

### Supplementary Information

**Additional file 1. Data comparison of PCV2 internalization into T-lymphoblasts.** This table compares the differences in virus internalization between the Landrace and Piétrain pigs for the six strains studied in the main text, in terms of the percentage of positive cells and average positive area (pixels).

**Additional file 2. Representative images of 200 nm red-fluorescent carboxylate-modified microspheres and green PCV2 particles immunostained with anti-PCV2-Cap 12E12 mAb.** This figure shows representative fluorescence images that are used to quantify the PCV2 particles based on the ratio between red fluorescent beads and green PCV2 particles. The density of the red beads is known and provided by the supplier.

**Additional file 3. Expression of CD3 on T-lymphoblasts from Landrace and Piétrain pigs.** All data were obtained from three pigs of each breed of T-lymphoblasts. The mean was calculated within each replicate based on ten randomly selected fields. (A) representative confocal images showing T-lymphoblasts stained in red with specific antibodies against CD3, a T cell marker. Nuclei were counter-stained with Hoechst 33342. (B) Quantification of the percentage of T-lymphoblasts expressing CD3. Data represent the means ± SD of three replicates. The Student’s *t*-test revealed the difference between T-lymphoblasts from the two breeds. This figure shows representative fluorescence images of CD3 expression on T-lymphoblasts and the percentages of CD3 + cells in the two breeds. There was no significant difference.

## Data Availability

The datasets generated and/or analysed during the current study are available from the corresponding author on reasonable request.

## Abbreviations

AEBSF: 4-(2-Aminoethyl) benzenesulfonyl fluoride hydrochloride
CatG: Cathepsin G
CCK-8: Cell Counting Kit 8
CQ: Chloroquine
CS: Chondroitin sulfate
DS: Dermatan sulfate
EE: Early endosome
FCS: Fetal calf serum
GAG: Glycosaminoglycan
HA: Hyaluronan
HS: Heparan sulfate
IF: Immunofluorescence
KS: Keratan sulfate
LE: Late endosome
LSCM: Laser-scanning confocal microscope
mAb: Monoclonal antibody
pAb: Polyclonal antibody
PBMC: Peripheral blood mononuclear cell
PCV2: Porcine circovirus type 2
PCVAD: Porcine circovirus associated disease
PDNS: Porcine dermatitis and nephropathy syndrome
PFA: Paraformaldehyde
PMWS: Postweaning multisystemic wasting syndrome
RT: Room temperature
