# Supplementary material for: Molecular basis for the different PCV2 susceptibility of T-lymphoblasts in Landrace and Piétrain pigs
Source: Vet Res. 2024 Feb 19;55:22. doi: 10.1186/s13567-024-01275-0 (PMC10875804; doi:10.1186/s13567-024-01275-0)
Supplement: Supplementary file 1 — Additional file 1. Data comparison of PCV2 internalization into T-lymphoblasts. This table compares the differences in virus internalization between the Landrace and Piétrain pigs for the six strains studied in the main text, in terms of the percentage of positive cells and average positive area (pixels). [file 13567_2024_1275_MOESM1_ESM.docx]

| **Additional file 1** **Data comparison of PCV2 internalization into T-lymphoblasts^a^** | | | | | | | | | | | | | | | |
| --- | --- | --- | --- | --- | --- | --- | --- | --- | --- | --- | --- | --- | --- | --- | --- |
|  |  | Percentage of PCV2^+^ cells (%) | | | | |  | Fluorescing area of PCV2 particles/PCV2^+^ cell (pixels) | | | | |  | Internalization ratio/PCV2^+^ cell (%)^b^ | |
|  |  | Surface sticking + Internalization | Solely internalization |  | Surface sticking + Internalization | Solely internalization |  | Surface sticking + Internalization | Solely internalization |  | Surface sticking + Internalization | Solely internalization |  |  |  |
| Strain |  | Landrace | |  | Piétrain | |  | Landrace | |  | Piétrain | |  | Landrace | Piétrain |
| Stoon-1010 (PCV2a) |  | 31.53 ± 17.08 | 13.98 ± 6.60 |  | 7.24 ± 1.84 | 2.82 ± 0.77 |  | 126 ± 30 | 53 ± 14 |  | 74 ± 9 | 45 ± 25 |  | 43.84 ± 10.09 | 58.75 ± 24.99 |
| 1121 (PCV2a) |  | 19.54 ± 3.65 | 5.67 ± 0.94 |  | 7.73 ± 1.19 | 2.11 ± 0.31 |  | 34 ± 15 | 22 ± 20 |  | 25 ± 7 | 22 ± 7 |  | 56.26 ± 31.62 | 89.03 ± 2.88 |
| 1147 (PCV2b) |  | 12.88 ± 1.87 | 3.01 ± 1.91 |  | 8.56 ± 0.62 | 1.36 ± 0.19 |  | 19 ± 15 | 16 ± 12 |  | 6 ± 0 | 5 ± 2 |  | 88.78 ± 10.72 | 83.33 ± 28.87 |
| 09V448 (PCV2d-1) |  | 15.30 ± 9.97 | 3.18 ± 1.61 |  | 8.62 ± 1.15 | 2.33 ± 0.32 |  | 30 ± 11 | 17 ± 12 |  | 12 ± 5 | 11 ± 6 |  | 53.44 ± 37.44 | 88.89 ± 19.25 |
| DE222-13 (PCV2d-2) |  | 74.61 ± 2.96 | 43.08 ± 3.33 |  | 57.45 ± 11.16 | 42.44 ± 7.18 |  | 521 ± 331 | 211 ± 159 |  | 307 ± 84 | 158 ± 23 |  | 38.08 ± 12.31 | 53.19 ± 10.10 |
| 19V245 (PCV2d-2) |  | 85.44 ± 1.53 | 57.30 ± 6.31 |  | 68.73 ± 14.03 | 48.71 ± 14.17 |  | 1502 ± 726 | 770 ± 232 |  | 624 ± 331 | 378 ± 211 |  | 54.55 ± 12.40 | 59.50 ± 8.65 |
| ^a^Data: Mean ± SD | | | | | | | | | | | | | | | |
| ^b^Internalization ratio (%) = internalized PCV2 particles/cell (pixels) ÷ total PCV2 particles/PCV2^+^ cell (pixels) × 100 | | | | | | | | | | | | | | | |
