# Supplementary material for: Molecular basis for the different PCV2 susceptibility of T-lymphoblasts in Landrace and Piétrain pigs
Source: Vet Res. 2024 Feb 19;55:22. doi: 10.1186/s13567-024-01275-0 (PMC10875804; doi:10.1186/s13567-024-01275-0)
Supplement: Supplementary file 2 — Additional file 2. Representative images of 200 nm red-fluorescent carboxylate-modified microspheres and green PCV2 particles immunostained with anti-PCV2-Cap 12E12 mAb. This figure shows representative fluorescence images that are used to quantify the PCV2 particles based on the ratio between red fluorescent beads and green PCV2 particles. The density of the red beads is known and provided by the supplier. [file 13567_2024_1275_MOESM2_ESM.docx]

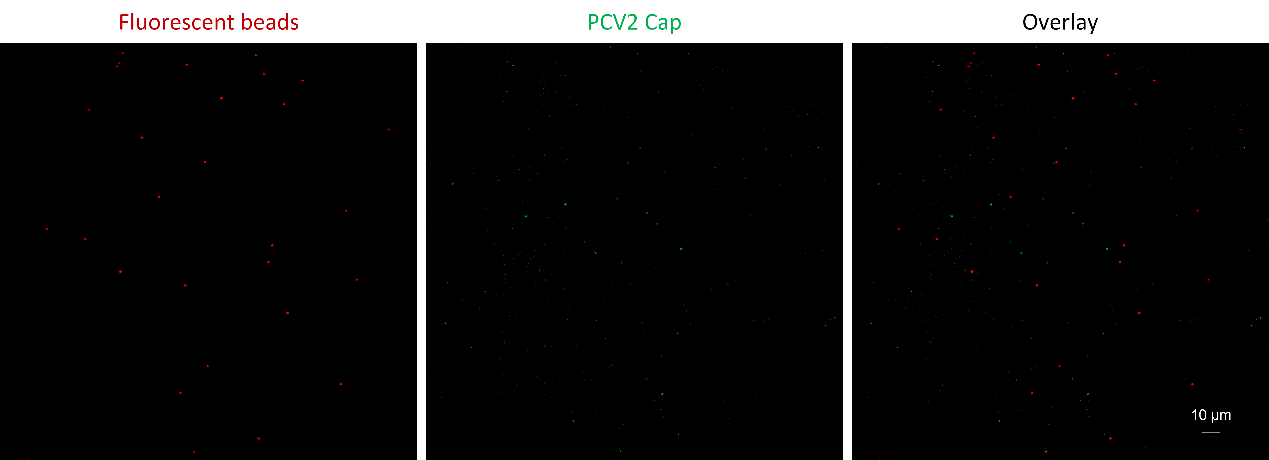


**Additional file 2. Representative images of 200 nm red-fluorescent carboxylate-modified microspheres and green PCV2 particles immunostained with anti-PCV2-Cap 12E12 mAb.** The images were obtained under identical conditions using confocal microscopy.
