# Supplementary material for: Molecular basis for the different PCV2 susceptibility of T-lymphoblasts in Landrace and Piétrain pigs
Source: Vet Res. 2024 Feb 19;55:22. doi: 10.1186/s13567-024-01275-0 (PMC10875804; doi:10.1186/s13567-024-01275-0)
Supplement: Supplementary file 3 — Additional file 3. Expression of CD3 on T-lymphoblasts from Landrace and Piétrain pigs. All data were obtained from three pigs of each breed of T-lymphoblasts. The mean was calculated within each replicate based on ten randomly selected fields. (A) representative confocal images showing T-lymphoblasts stained in red with specific antibodies against CD3, a T cell marker. Nuclei were counter-stained with Hoechst 33342. (B) Quantification of the percentage of T-lymphoblasts expressing CD3. Data represent the means ± SD of three replicates. The Student’s t-test revealed the difference between T-lymphoblasts from the two breeds. This figure shows representative fluorescence images of CD3 expression on T-lymphoblasts and the percentages of CD3 + cells in the two breeds. There was no significant difference. [file 13567_2024_1275_MOESM3_ESM.docx]

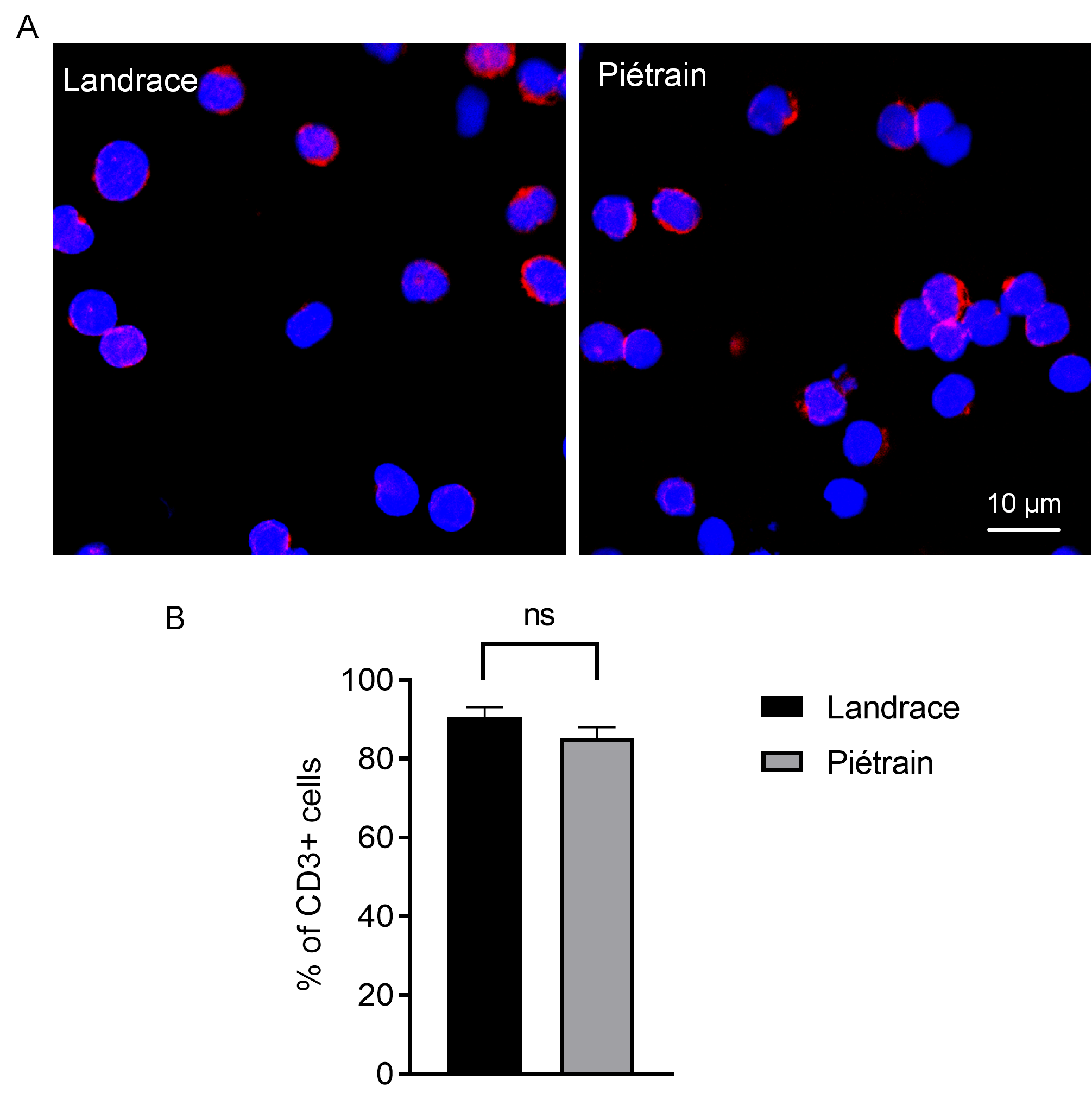


**Additional file 3 Expression of CD3 on T-lymphoblasts from Landrace and Piétrain pigs.** All data were obtained from three pigs of each breed of T-lymphoblasts. The mean was calculated within each replicate based on ten randomly selected fields. (A) representative confocal images showing T-lymphoblasts stained in red with specific antibodies against CD3, a T cell marker. Nuclei were counter-stained with Hoechst 33342. (B) Quantification of the percentage of T-lymphoblasts expressing CD3. Data represent the means ± SD of three replicates. The Student’s *t*-test revealed the difference between T-lymphoblasts from the two breeds.
